# RFA measurements of survival midpalatal orthodontic mini-implants in comparison to initial healing period

**DOI:** 10.1186/s40510-020-0305-x

**Published:** 2020-02-17

**Authors:** Manuel Nienkemper, Jan H. Willmann, Kathrin Becker, Dieter Drescher

**Affiliations:** 1 Department of Orthodontics, Heinrich-Heine-University of Düsseldorf, Moorenstr.5, Building 18.21, 40225 Düsseldorf, Germany; 2 Private Practice, Düsseldorf, Germany

**Keywords:** Mini-implants, Stability, Long term, Implant length

## Abstract

**Background:**

In dental implantology, the development of stability over time is a well-investigated topic. In case of orthodontic mini-implants, quantitative data for long-term stability is not available yet. This study aims to clinically investigate the long-term stability of mini-implants inserted in the midsagittal suture of the anterior palate. Moreover, the influence of the length of implants was elucidated. The stability of 2 × 9 and 2 × 11 mm mini-implants after orthodontic treatment (9 mm, 2.84 years ± 1.25 years; 11 mm, 3.17 years ± 0.96 years) was assessed by resonance frequency analysis (RFA). The obtained long-term pieces of data were compared with each other (9 mm vs 11 mm), as well as with the data from the matched early stability groups, to assess the initial and early secondary stability after the insertion from previous clinical trials.

**Results:**

For both lengths, the long-term stability (2 × 9 mm, 25.12 ± 7.11, *n* = 21; 2 × 11 mm, 24.39 ± 5.82, *n* = 18) was significantly lower than primary stability (2 × 9 mm, 36.14 ± 6.08, *n* = 19; 2 × 11 mm, 33.35 ± 3.53, *n* = 20). The differences within the groups disappeared over the initial healing period: after 4 weeks for the 2 × 9 mm implants and after 2 weeks for the 2 × 11 mm implants. Also, the 2 × 9 mm and 2 × 11 mm implants showed comparable long-term stability values.

**Conclusion:**

The stability of midpalatal mini-implants does not change in the long term after the initial healing period. Moreover, 2 × 9 mm mini-implants seem to be appropriate for orthodontic anchorage, as the stability of 2 × 11 mm implants is not higher. Therefore, owing to lower invasiveness, 2 × 9 mm implants should be preferred.

## Background

Skeletal anchorage gained popularity for expanding the biomechanical modalities of orthodontic treatment and expanding the scope of orthopaedic treatment. It can be accomplished with mini-plates, mini-implants (TADs), or orthodontic implants. Among these, mini-implants are favoured by many clinicians. The ease of clinical use, reasonable cost, easy insertion and removal, and the possibility of immediate loading based on sufficient primary stability were the reasons for their frequent use in orthodontic practice [1–5].

The survival rate of mini-implants in the anterior palate is reported to be 97.9% in contrast to interradiculary inserted mini-implants with a failure rate of 10–30% [6, 7]. Within the so-called T-Zone, the anterior palate offers bone with high quality and is covered with thin mucosa [8–11]. Nonetheless, until now, no quantitative data documents the long-term stability of mini-implants and how the stability develops over time. From in vivo studies in dental implantology, it is well known that dental implant stability is subject to changes up to 20 months after the insertion [12–16].

The gold standard technique to measure dental implant stability, namely resonance frequency analysis (RFA), was successfully transferred and established to measure the stability of mini-implants [13, 17–22]. So far, mini-implant stability in the anterior palate was followed for 6 weeks through examination of the transition for primary to early secondary stability [20–22]. To our best knowledge, the long-term stability of mini-implants using RFA had not been studied in vivo.

The mini-implant length (9 mm vs 11 mm) does not seem to affect primary and early secondary stability in the anterior palate [20]. Until now, no long-term data exists about the influence of the implant length on the long-term stability of mini-implants inserted in comparable areas in the anterior palate. Hence, the aim of this study was to investigate:

1. The stability of midpalatal orthodontic mini-implants after orthodontic treatment.
2. The influence of the implant length on the long-term stability of mini-implants inserted in the midsagittal suture of the anterior palate.
3. To compare the primary and early secondary stability data from previous prospective clinical trials with the RFA values prior to removal of the mini-implants.

It is hypothesized that the stability of the mini-implants can be attributed to the change after the initial healing period of 6 weeks and that the implant length has no significant impact on the long-term stability of the implants.

### Subjects and methods

The stability of 9-mm and 11-mm mini-implants after orthodontic treatment was assessed by RFA (long-term group 9—LT9, long-term group 11—LT11).

Pieces of data were compared with each other, as well as with the data from a matched initial healing period group (initial healing group 9—IG9, initial healing group 11—IG11), to assess the initial and early secondary stability of mini-implants in a repeated cross-sectional study design [20].

**Fig. 1:**
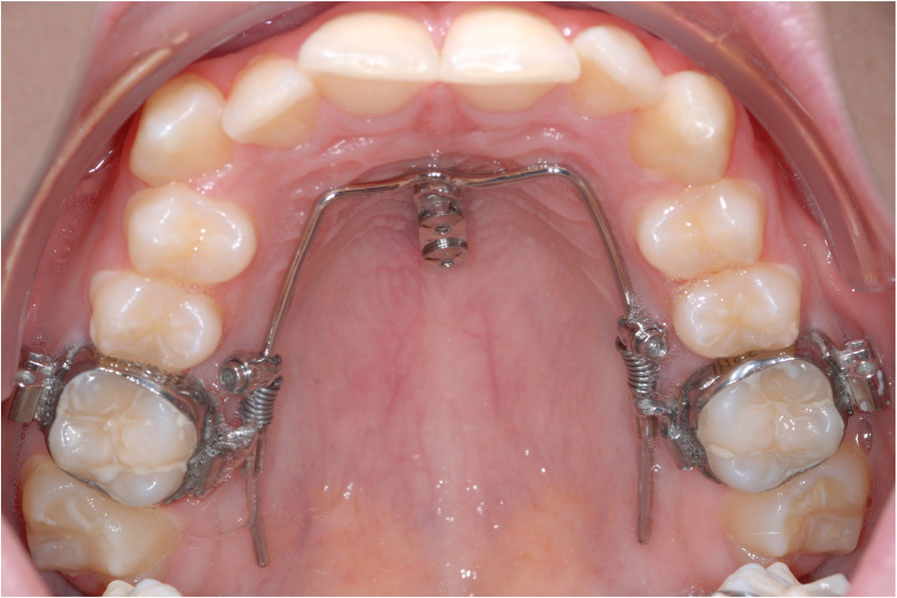
Orthodontic sliding mechanics for sagittal molar movement

All the patients that underwent treatment employing median mini-implants in the anterior palate of 2 × 9 mm or 2 × 11 mm (Benefit, PSM, Tuttlingen, Germany) were consecutively asked to participate in the study. The distance between the mini-implants is given by the connecting plate which is clinically ranged between 7 mm and 9 mm. Further inclusion criterion was the previous use of sliding mechanics for sagittal molar movement, 200 cN each side (Fig. 1). The implants and soft tissues were examined. Exclusion criteria were patients with systemic diseases affecting the bone metabolism, cleft patients, and patients showing signs of peri-implant inflammation. Visual inspection, performed before the removal of the implant, included detection of infection-related reddening and swelling. The tests for bleeding on probing were performed with a peri-odontal probe on four sites at each implant. Positive bleeding on probing without signs of any marginal bone loss around the implant was recorded as peri-mucositis. Informed consent was obtained from all the participants of this study. Therapeutic success was not a selection criterion.

Mini-implant insertion was carried out using a standardized protocol in all the groups. After predrilling with a burr of 1.3 mm in diameter to a depth of 3 mm, the mini-implants were inserted perpendicular to the palatal surface. The 2 × 9 mm mini-implants were inserted distal to the third ruga palatina, while the 2 × 11 mm implants were inserted slightly more anterior. The gingival thickness was measured using a dental probe after local anaesthesia. The appropriate gingival thickness was defined between 1 and 2 mm. The insertion and predrilling were performed using a surgical machine (ElcoMed SA 200C, W&H, Bürmoos, Austria). The identical exclusion criteria were applied to both groups. The study was conducted in accordance with the Declaration of Helsinki guidelines on experimentation involving human subjects and was approved by the local ethics committee.

**Fig. 2:**
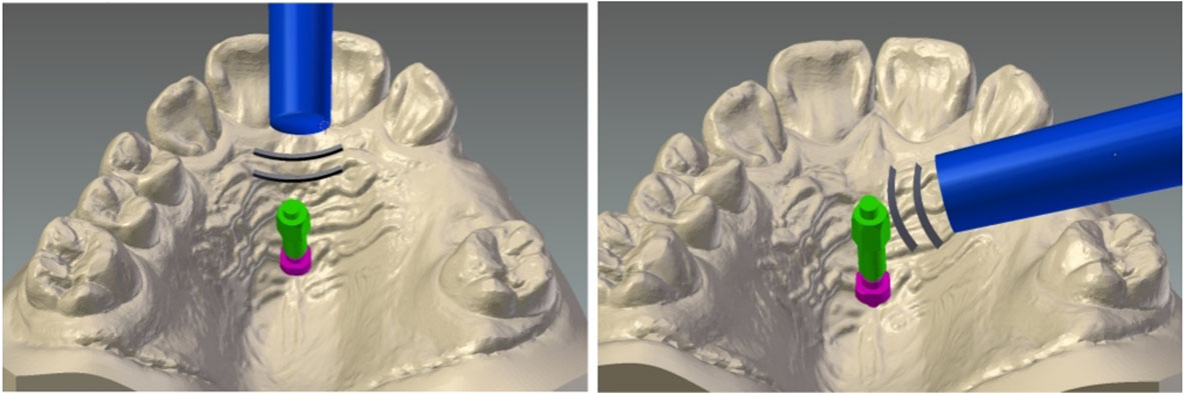
Measurement technique using the Osstell mentor. Left, parallel to the midpalatal suture. Right, perpendicular to the midpalatal suture

After the removal of the mechanics and prior to the removal of the implants (T4), RFA was performed using the Osstell ISQ device (Osstell, Gothenburg, Sweden):

- Three times parallel to the midpalatal suture
- Three times perpendicular to the midpalatal suture (Fig. 2)

The removal of the mini-implant was carried out manually using a manual-driven contra-angled hand piece without local or topical anaesthesia.

The data obtained from these patients was compared with the matching groups from the previous prospective clinical trials examining primary and early secondary of mini-implants [20]. In this study, the stability of 2 × 9 mm median (IG9) and 2 × 11 median (IG11) mini-implants was observed during the healing phase over a period of 6 weeks. Mini-implant stability was measured on four different occasions using RFA:

- T0—immediately after the insertion
- T1—2 weeks after the insertion
- T2—4 weeks after the insertion
- T3—6 weeks after the insertion

To ensure the comparability of the groups (IG9 + 11 and LT9 + 11), the mini-implant diameter, insertion site, insertion protocol, vertical bone height, and the orthodontic appliance were nearly identical.

In total, 78 implants were investigated in this study. The distribution of the sample in each group presented the following composition: LT9, *n* = 21; LT11, *n* = 18; IG9, *n* = 19; and IG11, *n* = 20.

In the LT11 group, two implants were dropped out due to signs of peri-mucositis. In the LT9 group, one implant was dropped out due to implant loosening with clinical signs of mobility. In the IG groups, three dropouts were reported respectively [20].

### Statistics

The sample size for the long-term groups was derived from a previous clinical trial [20]. Our data was compared with the data from that clinical trial. In addition, we retrospectively investigated the confidence intervals to confirm the relevance of the measurements (Table 3).

Group matching regarding age at the mini-implant insertion was tested with the Kruskal–Wallis test based on non-normal distribution (Shapiro–Wilk test). Gender matching was tested with the chi-square test. The vertical bone height was tested with a univariate ANOVA based on normal distribution (Shapiro–Wilk test).

The treatment time was compared using the Student’s *t* test for independent samples based on the normal distribution of the parameters (Shapiro–Wilk test).

Mean ISQ (implant stability quotient) values and standard deviations were calculated.

The ISQ values prior to the removal of the implants (2 × 9 mm and 2 × 11 mm) at T4 were compared with each other, as well as with the data from a previous prospective clinical trial examining primary and early secondary stability. Based on a test for normal distribution for small sample sizes (Shapiro–Wilk test), a univariate ANOVA was carried out to perform intra-group comparisons, followed by a Tukey post hoc test, wherever appropriate. Inter-group differences for each measurement time were tested with the Student’s *t* test for independent samples. The statistical analysis was carried out using SPSS Statistics 23 (IBM, Chicago, USA).

## Results

The mean treatment duration was 3.17 ± 0.96 years in the LT11 group compared with 2.84 ± 1.25 years in the LT9 group (Table 1).

**Table 1:** Descriptive statistics and matching of the groups

	IG11		LT11		IG9		LT9		Statistical test	Significance
Gender	F	M	F	M	F	M	F	M	Chi-square	0.696 n.s.
	10	10	6	12	8	11	12	9		
Age (years)	15.61 ± 6.96		16.77 ± 7.75		15.54 ± 7.31		16.21 ± 3.89		Kruskal–Wallis test	0.401 n.s.
Implants measured	20		18		19		21			
Treatment time (years)			3.17 ± 0.96				2.84 ± 1.25		*t* test	0.067 n.s.
Vertical bone height	4.87 ± 0.86		4.59 ± 0.87		5.28 ± 1.25		4.62 ± 1.10		ANOVA	0.237 n.s.

### Group matching

The mean treatment duration was 3.17 ± 0.96 years in the LT11 group compared with 2.84 ± 1.25 years in the LT9 group (Table 1). The treatment duration did not differ significantly between the groups. The statistical analysis revealed no difference regarding age and gender between the groups. The comparison of the vertical bone height revealed no significant differences (Table 1).

### Longitudinal results 2 × 11 median

**Fig. 3:**
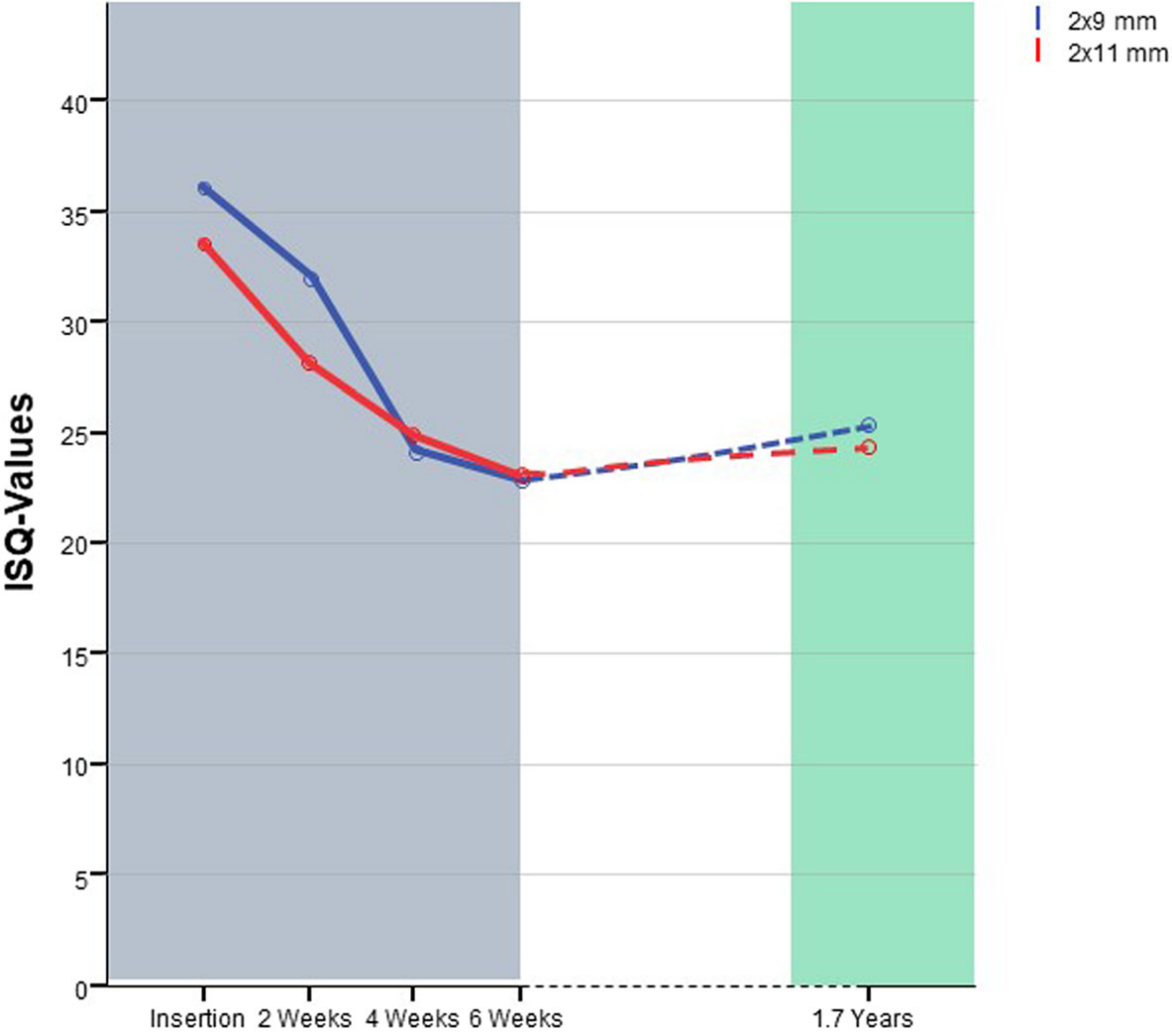
Development of stability for 2 × 9 and 2 × 11 mm implants

ANOVA revealed significant differences between the observational time points (Table 2). Primary stability (T0, 33.35 ± 3.53) was significantly higher than the stability prior to the explanation (T4, 24.39 ± 5.82). The comparison of T4 values with other time points (T1–T3) reveals no statistically significant differences (Table 3). The development of stability from T0 to T3 has been subject to prior studies [22]. The development of stability is shown in Fig. 3.

**Table 2:** Mean ISQ values: inter-group comparison (columns) and intra-group changes over time (rows)

	T0		T1		T2		T3		T4		ANOVA
	Mean	SD	Mean	SD	Mean	SD	Mean	SD	Mean	SD	
2 × 11 mm	33.35	3.53	28.1	3.99	24.63	4.46	22.9	6	24.39	5.82	< 0.001
											***
2 × 9 mm	36.14	6.08	32.11	5.57	24.23	7.19	22.51	6.69	25.12	7.11	< 0.001
Difference	− 2.79		− 4.01		0.4		0.39		0.73		***
Inter-group (*t* test)	0.087		0.013		0.834		0.987		0.729		
	n.s.		*		n.s.		n.s.		n.s		

*p < .05, **p < .001, ***p < .0001

**Table 3:** Results of Tukey post hoc test 2 × 11 and 2 × 9 intra-group comparison and respective confidence intervals (CI 95%)

	T0	T1	T2	T3
Post hoc 2 × 11				
T4	< 0.001***	0.134	1.000	0.879
CI 95%	− 13.35	− 8.1	− 4.63	− 2.81
	–	–	–	–
	− 4.6	0.65	4.12	5.85
Post hoc 2 × 9				
T4	< 0.001***	0.008**	0.993	0.823
CI 95%	− 16.73	− 12.70	− 4.82	− 3.52
	–	–	–	–
	− 5.31	− 1.29	6.6	7.9

*p < .05, **p < .001, ***p < .0001

### Longitudinal results 2 × 9 median

ANOVA revealed significant differences between the observational time points (Table 2). Primary stability (T0, 36.14 ± 6.08) and the stability 2 weeks after the insertion (T1, 32.1 ± 5.5) was significantly higher than the stability prior to the explanation (T4, 25.12 ± 7.11). The comparison of T4 values with time points (T2 and T3) reveals no statistically significant differences (Table 3). The detailed development of stability from T0 to T3 has been subject to prior studies [22]. The development of stability is shown in Fig. 3.

### Comparison of long-term stability (LT9 vs LT11)

The ISQ values in both groups are nearly identical (LT9, 25.11 ± 7.11/LT11: 24.3 ± 5.82). The comparison of T4 values between the two groups by using the Student’s *t* test for independent samples did not reveal a significant difference. The overall development of stability is shown in Fig. 3.

## Discussion

The success of mini-implant treatment depends on the relation between stability and loading. Any evaluation of the implant survival rate only gives information about the specific protocol (type of loading, insertion protocol) used in the respective study. If the clinician wants to plan his/her individual treatment and loading of the mini-implant, it is important for him/her to know the stability at any stage of the healing period. He/she needs to know how the stability changes over time and if there is a long-term change in the stability. This might affect the time and the amount of loading. Palatal mini-implants are often used nowadays for more than one purpose [1]*.* Especially in these cases, the mini-implants stay in use for an extended period after initial healing and even after the completion of one treatment goal.

One drawback of the study is that it is not based on longitudinal data from identical patients. To overcome this drawback, the initial stability groups and study groups were matched regarding age and gender, implant position, implant type, gingival thickness, measurement method, and vertical bone height. Of course, the vertical palatal bone height varies greatly among individuals. Therefore, the mini-implants were inserted in a region offering sufficient high-quality bone [9–11]. The literature recommends a T-shaped area posterior to the third palatal rugae as an optimal insertion site [9–11]. Since the groups showed no significant statistical differences regarding the parameters, an assessment of mini-implant stability over a long term was possible. Mini-implants with signs of a peri-implant inflammation were excluded because peri-mucositis may develop into peri-implantitis. Mini-implants with this type of progressing inflammation lose their stability and would not be suitable to determine long-term secondary stability.

Previous studies showed that during the first 4 weeks, mini-implant stability undergoes a significant change from primary to early secondary stability [20–22]. This decrease can be explained by remodelling taking place at the implant bone interface [12, 14, 15]. Interestingly, longer implants did not show higher primary and early secondary stability compared with shorter implants at the similar insertion spot in the anterior palate [22]. The implants examined in this study had a polished surface, and therefore, the main factor contributing to stability is macro-retention. Hence, one would assume that longer implants would lead to higher mechanical stability. This mechanical assumption, which was also previously considered in dental implantology, could not be supported by the data collected and examined in this study [23].

Over a comparable treatment period, the implant length (9 mm vs 11 mm) was not a significantly impacting mini-implant stability in the anterior palate. Hence, our hypothesis has to be rejected. RFA values in both LT groups at T4 were slightly higher than those at the IG groups at T3. However, this difference was not statistically significant. It seems that mini-implant stability is subject to change up to 4 weeks after the insertion and remains constant for the rest of the treatment. These findings are supported by previous evidence, which indicated main remodelling at the bone to mini-implant interface 2 to 4 weeks after the MI insertion [24–26].

In in vivo studies, examination of the stability of dental implants shows a typical ISQ curve representing initially decreasing stability followed by an increase [12, 13, 15, 27]. Studies by Friberg, Sennerby, and Guler showed that the stability of dental implants is subject to change for periods of up to 20 months regardless of the implant length and the insertion site [13, 15]. Interestingly, long-term follow-up measurements of the maxillary dental implants inserted in a region with a comparable bone density revealed that such implants tend to reach a similar level of stability irrespective of the initial stability [13]. The data from our study suggests that a comparable process seems to occur in the anterior palate and mini-implants. The average ISQ values of stable mini-implants and stable dental implants differ considerably (25 vs 60), which, besides the smaller size, can be explained with the smooth surface, resulting in a lower level of osseointegration [28]. In contrast to dental implants, mini-implant stability does not increase after initial healing. However, the remodelling at the implant-bone interaface seems to have continuously adapted to the forces applied to the implants, resulting in a balanced state.

The 2 × 11 mm implants were inserted slightly more anterior compared with the 2 × 9 mm group in a region with a thicker bone [29]. The results show that the position of the implants does not seem to affect the stability if the anterior limit of mini-implant insertion (third palatinal rugae) is respected [8, 11]. Implants placed close to the third rugae or even further anterior may have a higher risk of penetrating the canalis incisivus—this might damage the nasopalatine bundle [30, 31]. Unpublished data from a CBCT study with virtually inserted mini-implants shows that there is a penetration risk of 27.9% for median insertion. The further the posterior insertion, the less the risk of penetration. Even though the percentage seems quite high, the low number of sequelae from such penetration, such as numbness of the anterior palatal mucosa from damaging the nasopalatinal nerve or non-osseointegration, might be explained with the idea of easily slipping small fibres within the lumen of the canal and its high anatomic variability [32, 33].

Also, 2 × 9 mm and 2 × 11 mm implants provide equivalent stability in both short and long terms. So, the second hypothesis has to be rejected as well. Since treatment was successful in the examined patients, one may conclude that RFA values of 22 to 25 offer a level of stability that is suitable for orthodontic purposes if the healing phase is survived. Our finding, as suggested by the studies by Sarul and Gracco, indicates that a length of 9 mm seems appropriate for orthodontic anchorage and easy removal after treatment [34, 35].

In complex cases, the stability and reliability level of the mini-implants allows accomplishment of multiple treatment goals sequentially.

The success of mini-implant treatment depends on the quotient between stability and loading. The evaluation of the success rate provides half the information. For the clinician, it is important how the stability changes over time and if there is a long-term change in the stability. This might affect the time of loading and the amount of loading as palatal mini-implants are used nowadays for more than one purpose consecutively (1). In these cases, mini-implants stay in use for an extended period after initial healing.

## Conclusions

The stability of midpalatal mini-implants does not change in the long term after the initial healing period. Also, 2 × 9 mm mini-implants seem appropriate for orthodontic anchorage as the stability of 11-mm implants was not higher. In the anterior palate, shorter implants (9 mm vs 11 mm) with an equal diameter can be regarded as less invasive and therefore should be preferred over longer implants.

## Data Availability

Please contact author for data request.

## Abbreviations

IG11: Initial group 11-mm implants
IG9: Initial group 9-mm implants
ISQ: Implant stability quotient
LT11: Long-term group 11-mm implants
LT9: Long-term group 9-mm implants
RFA: Resonance frequency analysis
